# Autoimmunity-Related Risk Variants in PTPN22 and CTLA4 Are Associated With ME/CFS With Infectious Onset

**DOI:** 10.3389/fimmu.2020.00578

**Published:** 2020-04-09

**Authors:** Sophie Steiner, Sonya C. Becker, Jelka Hartwig, Franziska Sotzny, Sebastian Lorenz, Sandra Bauer, Madlen Löbel, Anna B. Stittrich, Patricia Grabowski, Carmen Scheibenbogen

**Affiliations:** ^1^Institute of Medical Immunology, Charité-Universitätsmedizin Berlin, Corporate Member of Freie Universität (FU) Berlin, Humboldt-Universität zu Berlin and Berlin Institute of Health (BIH), Berlin, Germany; ^2^Carl-Thiem-Klinikum Cottbus gGmbH, Research Center, Cottbus, Germany; ^3^BIH Center for Regenerative Therapies, Charité-Universitätsmedizin Berlin, Berlin, Germany; ^4^Labor Berlin—Charité Vivantes GmbH, Berlin, Germany

**Keywords:** single nucleotide polymorphism (SNP), tyrosine phosphatase non-receptor type 22 (PTPN22), cytotoxic T-lymphocyte-associated protein 4 (CTLA4), interferon regulatory factor 5 (IRF5), tumor necrosis factor (TNF), myalgic encephalomyelitis (ME), chronic fatigue syndrome (CFS), autoimmunity

## Abstract

Single nucleotide polymorphisms (SNP) in various genes have been described to be associated with susceptibility to autoimmune disease. In this study, myalgic encephalomyelitis/chronic fatigue syndrome (ME/CFS) patients and controls were genotyped for five immune gene SNPs in tyrosine phosphatase non-receptor type 22 (*PTPN22*, rs2476601), cytotoxic T-lymphocyte-associated protein 4 (*CTLA4*, rs3087243), tumor necrosis factor (*TNF*, rs1800629 and rs1799724), and interferon regulatory factor 5 (*IRF5*, rs3807306), which are among the most important risk variants for autoimmune diseases. Analysis of 305 ME/CFS patients and 201 healthy controls showed significant associations of the *PTPN22* rs2476601 and *CTLA4* rs3087243 autoimmunity-risk alleles with ME/CFS. The associations were only found in ME/CFS patients, who reported an acute onset of disease with an infection (*PTPN22* rs2476601: OR 1.63, CI 1.04–2.55, *p* = 0.016; *CTLA4* rs3087243: OR 1.53, CI 1.17–2.03, *p* = 0.001), but not in ME/CFS patients without infection-triggered onset (*PTPN22* rs2476601: OR 1.09, CI 0.56–2.14, *p* = 0.398; *CTLA4* rs3087243: OR 0.89, CI 0.61–1.30, *p* = 0.268). This finding provides evidence that autoimmunity might play a role in ME/CFS with an infection-triggered onset. Both genes play a key role in regulating B and T cell activation.

## Introduction

With an estimated prevalence of 0.2–0.3%, myalgic encephalomyelitis/chronic fatigue syndrome (ME/CFS) is a frequent and severe chronic multisystemic disease. Patients suffer from persistent exhaustion, cognitive dysfunctions, pain and flu-like symptoms, leading to a substantial reduction of life quality (1). The underlying pathomechanism is not well-understood, but there is convincing evidence that, at least in a subset of ME/CFS patients, autoimmunity contributes to disease etiology (2, 3). Autoantibodies against various antigens, including neurotransmitter receptors, were reported by several groups (3). Comorbidity with Hashimoto's thyroiditis, fibromyalgia, postural orthostatic tachycardia syndrome and a higher rate of autoimmune disease were reported for ME/CFS patients and their families (2, 3).

Enhanced immune activation and impairment of immunological tolerance are considered as important risk factors for autoimmunity. Autoimmune diseases are understood as being multifactorial with involvement of genetic and environmental factors. Disease onset is often triggered by infections, and the link between infections and autoimmune diseases is well-established (4). In recent years, genome-wide association studies have revealed single nucleotide polymorphisms (SNPs) in various genes to be associated with the risk to develop autoimmune diseases. For most of these SNPs, it was found that they confer gain- or loss-of-function in enzymes or transcription factors that play a role in B and T cell activation or cytokine production and cytokine signaling, which are crucial mechanisms for the development of autoimmune diseases (5–8). Among the most frequent variants associated with multiple autoimmune diseases are the tyrosine phosphatase non-receptor type 22 (*PTPN22*) SNP rs2476601, the cytotoxic T-lymphocyte-associated protein 4 (*CTLA4*) SNP rs3087243, the interferon regulatory factor 5 (*IRF5*) SNP rs3807306, and the SNP rs1800629 in the gene tumor necrosis factor (*TNF*).

The *PTPN22* SNP rs2476601 is one of the most important susceptibility loci for autoimmunity and is associated with myasthenia gravis (MG), type 1 diabetes (T1D), systemic lupus erythematosus (SLE), rheumatoid arthritis (RA), and other autoimmune disorders (9). This SNP was shown to play an important role in T and B cell receptor signaling (5). The SNP rs3087243 in *CTLA4* is associated with several autoimmune diseases, including SLE, RA, T1D, and Graves' disease (8, 10–14). The *CTLA4* SNP has a protective effect, and the major allele G is associated with autoimmunity. The G allele leads to an impaired negative regulation of T cell activation. Within multiple sclerosis (MS) patients, homozygous carriers of the G allele were shown to express significantly lower CTLA4 protein levels in CD4^+^ T cells (15). A SNP in *IRF5*, rs3807306, is another common autoimmune susceptibility locus associated with RA, SLE, MS, and Crohn's disease (CD) and is linked to higher serum interferon-α (IFNα) activity (measured by a reporter cell line) in SLE (7, 16–18). The SNP rs1800629 in *TNF* is linked to various autoimmune diseases, including CD, celiac disease, RA, and SLE (6, 19–21). Another SNP in the *TNF* gene rs1799724 is associated with CD and autoimmune hepatitis (AIH) (22, 23). Higher serum levels of TNFα were shown in carriers of the *TNF* rs1799724 risk genotype, while for the SNP rs1800629, data on an association with elevated TNFα serum levels are inconclusive (6, 24).

A recently published systematic review of Wang et al. summarizes the studies of genetic variants, which are associated with ME/CFS (25). Several SNPs in cytokines and human leukocyte antigen (HLA) associations were found in ME/CFS. Two SNPs in *TNF*, rs1800629 (TNF-308) and rs1799724 (TNF-857), were comparatively analyzed between a ME/CFS and control cohort (26). A higher frequency of the TNF-857 T allele was found in ME/CFS. Analysis of genetic variants in *PTPN22, CTLA4*, and *IRF5* were not described in ME/CFS so far.

In the present study, we comparatively analyzed the prevalence of the five SNPs described above in ME/CFS patients and healthy controls. ME/CFS onset is triggered by an infection in approximately two thirds of patients (27). The link between infections as a trigger of autoimmune diseases is well-established (4). Therefore, we also studied a possible association of these SNPs with an infection-triggered disease onset (ITO).

## Materials and Methods

### Patients and Controls

ME/CFS patients were diagnosed at the outpatient clinic for immunodeficiencies at the Institute for Medical Immunology at the Charité-Universitätsmedizin Berlin, Germany. Diagnosis of ME/CFS is based on Canadian Consensus Criteria (CCC) (28) and exclusion of other medical or neurological diseases that may cause fatigue. Autoimmune diseases were an exclusion criterion, with the exception of Hashimoto's thyroiditis, which is classified as comorbidity in ME/CFS (1). Controls not suffering from fatigue were recruited from staff. Characteristics of patients are shown in Table 1. 232 of 305 patients stated that they had an acute onset of illness with an infection (ITO). We retrospectively collected the data of the types of infection from the patients records and could classify most patients into the categories “respiratory or gastrointestinal tract infection,” “primary EBV,” or “history of viral or bacterial infection.” All patients and controls were caucasian. The study was approved by the Ethics Committee of Charité-Universitätsmedizin Berlin in accordance with the 1964 Declaration of Helsinki and its later amendments (EA4-090-10). All patients and healthy controls recruited from staff gave written informed consent.

**Table 1:** Cohort characteristics.

**ME/CFS**			
	**All**	**w/ ITO**	**w/o ITO**
	***n*** **= 305**	***n*** **= 232**	***n*** **= 73**
Age [median (range)]	44 (18–75)	42 (18–71)	50 (19–75)
Gender distribution female/male [number]	205/100	158/74	47/26
Bell Score [median (range)]	30 (10–70)	30 (10–70)	38 (10–70)
Elevated TPO antibody^*^>34 kU/l	8% (*n* = 158)	6% (*n* = 120)	13% (*n* = 38)
Elevated ANA >1:160	19% (*n* = 225)	20% (*n* = 163)	16% (*n* = 62)
**Acute onset of disease with an infection [%]**			
Respiratory tract		26	
Viral		22	
Primary EBV		19	
Bacterial		12	
Gastrointestinal tract		5	
Not specified		16	
**HEALTHY CONTROLS**			
	**All**		
	***n*** **= 201**		
Age [median (range)]	29 (19–65)		
Gender distribution female/male [number]	103/98		

*ITO, infection triggered onset; w/, with; w/o, without; TPO, hyreoperoxidase antibodies; ANA, Antinuclear antibodies; EBV, Epstein-Barr virus*.

* *not significant*.

### DNA Extraction and Allelic Discrimination PCR

We analyzed SNPs in genomic coding regions described as risk factors for autoimmunity (Table 2) by allelic discrimination (AD) polymerase chain reaction (PCR). Genomic DNA from patients and healthy controls was obtained from whole blood samples or peripheral blood mononuclear cells (PBMCs) using the QIAmp DNA Blood Mini Kit (QIAGEN) and stored at −20°C in nuclease-free water until use. DNA was quantified using NanoDrop™ spectrophotometer. Genotyping of all five SNPs (*PTPN22* rs2476601, ID: C__16021387_20; *CTLA4* rs3087243, ID: C___3296043_10; *TNF* rs1800629, ID: C___7514879_10; *TNF* rs1799724, ID: C__11918223_10; and *IRF5* rs3807306, ID: C___2691231_10) was performed using pre-designed TaqMan allelic discrimination assay probes (Thermo Fisher). The 10 μl PCR reactions contained 2 μl of template DNA (5 ng/μl), 0.5 μl of the SNP genotyping Assay Mix, 5 μl of TaqMan Universal Mastermix II no UNG (Applied Biosystems), and 2 μl sterile distilled water. PCR run was performed in a 7500 Fast & 7500 Real-Time PCR System (Applied Biosystems). Background fluorescence was recorded performing a pre-read run for 1 min at 60°C. Amplification was achieved through activation of DNA polymerase for 10 min at 95°C, following 40 cycles of melting for 15 s at 92°C and annealing/extension for 1 min at 60°C. Afterwards, background fluorescence was automatically subtracted from the amplification results by running a post-read for 1 min at 60°C. AD PCR results were visualized in a cluster plot of allele X vs. allele Y to distinguish between homo- and heterozygosity.

**Table 2:** SNPs associated with autoimmune diseases and their functional effect.

**SNP**	**Gene**	**Disease association**	**Functional effect**
rs2476601 A>G	*PTPN22*	RA, SLE, T1D, GD, MG (9)	Modulation of T and B cell receptor signaling (29)
rs3087243 G>A	*CTLA4*	RA (11), SLE (12), T1D (13)	Negative regulation of T cell activation impaired (14)
rs1800629 G>A	*TNF*	CD (19), Celiac disease (20), GD, MS (30), RA, SLE (21)	Higher levels of TNFα (6)
rs1799724 C>T	TNF	CD (22), ME/CFS (26), Vitiligo (24), AIH (23)	Higher levels of TNFα (24)
rs3807306 G>T	*IRF5*	RA (16), SLE (17), MS (7), CD (18)	High serum IFNα activity (17)

*PTPN22, tyrosine-protein phosphatase non-receptor type 22; CTLA4, cytotoxic T-lymphocyte-associated protein 4; TNF, tumor necrosis factor; IRF5, interferon regulatory factor 5; AIH, autoimmune hepatitis; CD, Crohn‘s disease; GD, Grave‘s disease; MG, Myasthenia Gravis; MS, Multiple sclerosis; RA, rheumatoid arthritis; SLE, systemic lupus erythematosus; T1D, Type 1 diabetes; UC, Ulcerative colitis*.

### Assessment of Soluble Immune Marker and Lymphocytes

Lymphocyte phenotyping by flow cytometry including CD3^+^, CD4^+^, and CD8^+^ T cells and CD19^+^ B cells and serum protein analysis including C reactive protein (CrP), complement proteins C3 and C4, soluble IL-2 receptor (IL-2R), IgG, and TNFα in LPS-stimulated whole blood cells was carried out by Labor Berlin, Charité Vivantes GmbH, Berlin.

### Statistical Analysis

Allele distribution was analyzed using the additive model in a 2 × 2 and genotype distribution using a 2 × 3 contingency table and tested for significance by χ^2^-square test. One-tailed *t*-tests were performed to test for increased frequency of the known autoimmunity risk alleles in ME/CFS patients. Odds ratio (OR) and 95% confidence interval (CI) were calculated from the same contingency tables.

The Hardy-Weinberg equilibrium (HWE) was tested using a 2 × 3 χ^2^ table comparing the expected and observed genotypes of each SNP. No significant deviation from HWE was observed for any of the studied SNPs in patients or controls. The five SNP loci analyzed, reside on different chromosomes (*PTPN22* rs2476601 on chr1, *CTLA4* rs3087243 on chr2, *TNF* SNPs rs1799724 and rs1800629 on chr6, and *IRF5* rs3807306 on chr7) therefore, a physical linkage can be excluded.

Analysis of association of serum proteins and immune cells with onset type or *PTPN22* rs2476601 and *CTLA4* rs3087243 risk variants were analyzed using the two-tailed Mann–Whitney *U* test.

Statistical tests were performed using GraphPad Prism software (Version 6.00) or R (31). A *p* ≤ 0.05 was considered statistically significant.

## Results

### Analysis of Allelic and Genotype Distribution

We analyzed a cohort of 305 ME/CFS patients and 201 healthy controls for potential disease association of the *PTPN22, CTLA4, TNF*, and *IRF5* SNPs that were described to be risk loci for various autoimmune diseases (Table 2 and Supplementary Table 1).

We found a significantly increased frequency of the *PTPN22* rs2476601 risk allele A, that confers susceptibility to autoimmunity, in ME/CFS patients (allele frequency (AF) 12%, odds ratio (OR) 1.50, *p* = 0.033) compared to healthy controls (AF 8%, *n* = 201; Table 3). When subgrouping patients according to the type of onset of disease, we observed the significant association of the *PTPN22* rs2476601 risk allele A only in patients with ITO (AF 13%, OR 1.63, *n* = 232, *p* = 0.016), but not in those without ITO (AF 9%), compared to healthy controls (AF 8%).

**Table 3:** Allele frequencies and genotypes.

**Gene**	**SNP**	**Cohorts ME/CFS all n = 305 ME/CFS w/ ITO n =232 ME/CFS w/o ITO n = 73 controls n = 201**	**Genotype counts Mi/Het/Ma**	**Risk allele freq**.	***p*-value control vs. ME/CFS**	**OR (95% CI)**
*PTPN22*	rs2476601 G>A	ME/CFS all	2/68/235	0.12	**0.033**	1.50 (0.97–2.31)
		ME/CFS w/ ITO	1/57/174	0.13	**0.016**	1.63 (1.04–2.55)
		ME/CFS w/o ITO	1/11/61	0.09	0.398	1.09 (0.56–2.14)
		Controls	2/29/170	0.08		
*CTLA4*	rs3087243 G>A	ME/CFS all	36/155/114	0.63	**0.012**	1.34 (1.04–1.73)
		ME/CFS w/ ITO	23/112/97	0.66	**0.001**	1.53 (1.17–2.03)
		ME/CFS w/o ITO	13/43/17	0.53	0.268	0.89 (0.61–1.30)
		Controls	38/102/61	0.56		
*IRF5*	rs3807306 G>T	ME/CFS all	74/140/91	0.47	0.136	0.87 (0.67–1.17)
		ME/CFS w/ ITO	63/102/67	0.49	0.318	0.94 (0.72–1.23)
		ME/CFS w/o ITO	11/38/24	0.41	**0.023**	0.68 (0.46–0.99)
		Controls	50/104/47	0.51		
*TNF*	rs1800629 G>A	ME/CFS all	6/77/222	0.15	0.319	0.92 (0.65–1.31)
		ME/CFS w/ ITO	5/56/171	0.14	0.275	0.89 (0.61–1.30)
		ME/CFS w/o ITO	1/21/51	0.16	0.491	1.01 (0.60–1.70)
		Controls	4/55/142	0.16		
*TNF*	rs1799724 C>T	ME/CFS all	5/52/248	0.10	0.096	0.77 (0.52–1.14)
		ME/CFS w/ ITO	5/41/186	0.11	0.204	0.84 (0.56–1.27)
		ME/CFS w/o ITO	0/11/62	0.08	**0.043**	0.55 (0.28–1.10)
		Controls	2/47/150	0.13		

*PTPN22, tyrosine-protein phosphatase non-receptor type 22; CTLA4, cytotoxic T-lymphocyte-associated protein 4; TNF, tumor necrosis factor; IRF5, interferon regulatory factor 5; Mi, minor; Het, heterozygous; Ma, major; freq., frequency; ITO, infection triggered onset; w/, with; w/o, without; OR, odds ratio; CI, confidence interval. Bold values indicate statistically significant p-values (p ≤ 0.05)*.

Equally, the frequency of the *CTLA4* rs3087243 risk allele G is significantly higher in ME/CFS patients (AF 63%, OR 1.34), compared to healthy controls (AF 56%; Table 3). Again, we observed a significant association of the CTLA4 rs3087243 risk allele G only in patients with ITO (AF = 66%, OR = 1.53, *p* = 0.001), but not in those wthout ITO (AF 53%) compared to healthy controls (AF 56%).

Oppositely, the AF of the *IRF5* rs3807306 risk allele T was significantly lower in ME/CFS patients without ITO (AF 41%, OR 0.68, *p* = 0.023), compared to healthy controls (AF 51%; Table 3). No difference in the risk AF of the *TNF* rs1800629 SNP between patients and controls was observed, but the AF of the *TNF* rs1799724 risk allele T again was significantly lower in ME/CFS patients without ITO (AF 8%, OR 0.55, *p* = 0.043), compared to healthy controls (AF 13%, Table 3). No associations with *IRF5* or *TNF* was found in patients with ITO.

**Figure 1:**
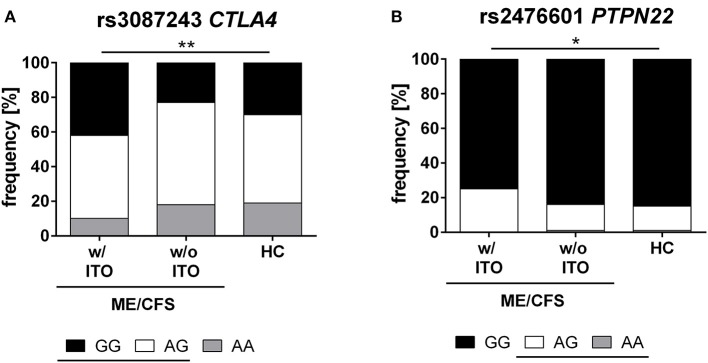
Genotype distribution of *CTLA4* and *PTPN22* SNPs. Distribution of major (black), hetero (white), and minor (gray) genotypes of *CTLA4* rs3087243 **(A)** and *PTPN22* rs2476601 **(B)** in ME/CFS patients with (*n* = 232) and without infection triggered onset (*n* = 73), and healthy controls (*n* = 201). The risk allele G in rs3087243 and A in rs2476601 are underlined. Statistical analyses were performed using 2 × 3 contingency tables and χ^2^- test. A *p* ≤ 0.05 was considered as statistically significant. HC, healthy control; w/ ITO, with infection-triggered onset; w/o ITO, without infection-triggered onset.

Further, we analyzed the genotype distribution for *CTLA4* rs3087243 and *PTPN22* rs2476601 (Figure 1). We found a significant difference between the ME/CFS patients with an ITO, compared to healthy controls (*CTLA4* rs3087243: *p* = 0.0061, *PTPN22* rs2476601: *p* = 0.026). ME/CFS patients without ITO had a similar genotype distribution as healthy controls.

### Association of Infection-Triggered Onset and PTPN22 and CTLA4 Variants With Immune Markers

**Figure 2:**
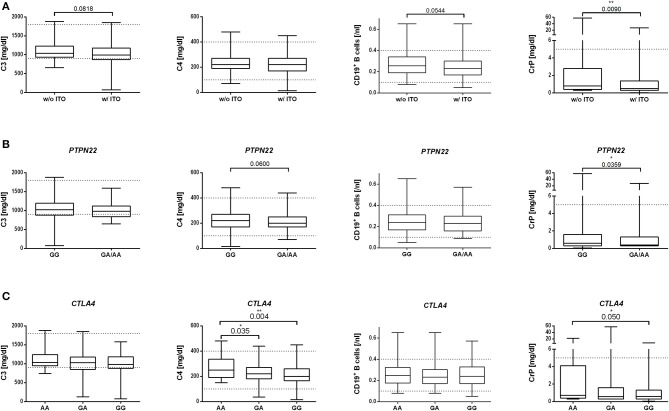
Association of disease onset type **(A)**, *PTPN22* SNP **(B)**, or *CTLA4* SNP **(C)** in ME/CFS patients with immune marker. Median with range of C3 and C4 complement levels [mg/dl], CD19+ B cells [/nl], and CrP [mg/dl] is shown for the ME/CFS subgroups **(A)** with (w/) or without (w/o) infection-triggered onset (ITO). **(B)** without or with *PTPN22* rs2476601 risk allele A or **(C)** without or with *CTLA4* rs3087243 risk allele G. Statistical analysis was performed using Mann–Whitney *U* test. A *p* ≤ 0.05 was considered as statistically significant.

We comparatively analyzed numbers of CD3^+^, CD4^+^, and CD8^+^ T cells and CD19^+^ B cells, CrP, complement proteins C3 and C4, soluble IL-2R, IgG, and TNFα in ME/CFS patients with and without ITO. Patients with ITO had lower CD19^+^ B cell levels (*p* = 0.05) and a trend toward lower C3 levels (Figure 2A and Supplementary Table 2). In patients with both, the risk allele in *PTPN22* rs2476601 (Figure 2B and Supplementary Table 3) or the homozygous risk allele for *CTLA4* rs3087243 (Figure 2C and Supplementary Table 4), C4 levels (*p* = 0.06 and 0.004, respectively) but not C3 levels were decreased, while there was no difference in B cell numbers.

CrP concentrations were significantly higher in ME/CFS patients without ITO (Figure 2A), and without the risk alleles of *PTPN22* rs2476601-A (Figure 2B) and *CTLA4* rs3087243-G (Figure 2C and Supplementary Tables 2–4).

CD3^+^, CD4^+^, and CD8^+^ T cells and soluble IL-2R, TNFα, and IgG concentrations did not differ between patient subgroups and risk variants (Supplementary Tables 2–4).

## Discussion

In this study, we evaluated five important SNPs that are associated with various autoimmune diseases in ME/CFS patients. Patients selected are representative of ME/CFS cohorts reported by other groups (with two-thirds of patients being female and reporting an ITO and an average disease severity of a Bell score of 30) (27). In accordance with this recent US study, ME/CFS onset in our patients was triggered most frequently by an acute respiratory infection or suspected viral infection (27). Also, a primary EBV infection in adolescence is a well-known trigger of ME/CFS and was reported in 19% of our patients. A primary EBV infection in adulthood is known as a risk factor for various autoimmune diseases (32).

We found a strong association of the autoimmunity risk alleles in *CTLA4* rs3087243-G and *PTPN22* rs2476601-A with ITO ME/CFS. Carrying the *PTPN22* rs2476601 risk allele A increased the odds 1.6-fold for developing infection-triggered ME/CFS. Carriers of the risk allele G of *CTLA4* rs3087243 had 1.5-fold-higher odds of developing ITO ME/CFS. Accordingly, the genotype distribution for both SNPs was significantly different in ME/CFS patients with ITO compared to healthy controls (Figure 1).

Both, the *PTPN22* rs2476601 and *CTLA4* rs3087243 SNPs were shown to be associated with numerous autoimmune diseases, including T1D, SLE, and RA (8–10, 33). *PTPN22* encodes a lymphoid tyrosine phosphatase that acts as a strong negative regulator of T cell activation. A study by Vang et al. provides evidence that the autoimmune risk allele A of *PTPN22* rs2476601 confers gain-of-function. This may lead to lower T cell receptor signaling and the inability to delete autoreactive T cells or diminished activity of Tregs (5). Further, the *PTPN22* rs2476601 risk allele may contribute to the generation of autoreactive B cells via disturbances in clonal deletion and receptor editing thus leading to impaired central tolerance (29). This is supported by the finding that the strongest associations with this SNP are found in autoimmune diseases in which autoreactive T cells and autoantibodies have a major role in the pathogenesis of the disease (29). In contrast, autoimmune diseases in which Th17 cells play an important role, and those of mucosal sites, tend to have no association with *PTPN22* (29).

*CTLA4* is expressed on CD4^+^ and CD8^+^ T cells upon activation and has a crucial role as negative T cell regulator to switch-off activated T cells (34). The risk allele G of rs3087243 leads to reduced levels of soluble *CTLA4* mRNA, which results in enhanced T cell activity (10, 15, 33).

Similar to our findings, both the *PTPN22* rs2476601 and *CTLA4* rs3087243 risk variants were shown to be susceptibility loci for autoimmune diseases including anti-neutrophil cytoplasmic antibody (ANCA)-associated vasculitis (AAV) and autoimmune polyglandular autoimmunity (35, 36). To our knowledge, the frequency of these SNPs has not been studied in other ME/CFS patients yet.

We further correlated our SNP data with immune markers. In patients with both the risk allele in *PTPN22* rs2476601 or the homozygous risk alleles for *CTLA4* rs3087243, C4 but not C3 levels were decreased. An association of these risk variants with C4 levels is not described in the literature. Low C4 levels are, however, frequent in SLE (37), a disease which is associated with both the risk allele in *PTPN22* and *CTLA4*.

When comparing patient subgroups, only those with ITO had diminished CD19^+^ B cells. B cell lymphocytopenia is a frequent finding in SLE and RA and provides further evidence for an autoimmune mechanism in ME/CFS patients with ITO (38, 39). In contrast, levels of CrP were significantly higher in ME/CFS patients without ITO and in those without the risk alleles. This may suggest that, within the subgroup of ME/CFS patients without ITO, there is a tendency to a more pro-inflammatory response similar to patients with cancer-related fatigue (40).

For the *IRF5* rs3807306 risk allele T we observed a significantly lower AF in ME/CFS patients without ITO compared to controls. This finding is unexpected and needs to be confirmed. We could not detect a difference in the frequency of the *TNF* rs1800629 AF between ME/CFS patients and healthy controls, but we again found a significantly lower AF of the TNF rs1799724 in ME/CFS patients without ITO compared to controls. This finding is in contrast to the previous study by Carlo-Stella et al., analyzing 80 ME/CFS patients, which found a higher frequency of the TNF-857 rs1799724 risk allele (26). We have no obvious explanation for this difference. The patient cohort in the 2006 study was diagnosed according to Fukuda criteria, which are less strict than the CCC criteria used in our study. Interestingly, these SNPs are associated with enhanced serum levels of TNFα and IFNα and also with autoimmune diseases of mucosal sites (19, 41). It is tempting to speculate that patients, who can produce higher levels of TNFα and IFNα, have a more effective mucosal immunity and are therefore less prone to develop ME/CFS. This is in line with our previous observation, that a deficiency of the complement factor mannose binding lectin (MBL), which results in susceptibility to infections, is associated with ME/CFS (42).

There are other variants of immune or immune-related genes described in ME/CFS as recently summarized (25). Several SNPs in HLA class II region were found to be associated with ME/CFS (43, 44). This association is another evidence for an autoimmune pathomechanism, as the link between certain HLA alleles and autoimmunity is well-established (45). Moreover, there are studies showing an association with several SNPs in the glucocorticoid receptor gene Nuclear Receptor Subfamily 3 Group C Member 1 (*NR3C1*). This receptor plays a role in the regulation of the inflammatory response by affecting the hypothalamic–pituitary–adrenal (HPA) axis activity trough cortisol release (30, 46). For ME/CFS, a deregulated HPA axis has been reported (47). In a similar manner, SNP variants in the catechol-O-methyltransferase (COMT) gene and the ß2 adrenergic receptor described in ME/CFS may modulate the immune response (48). Further studies found associations with SNPs not related to the immune system, like variants associated with muscle metabolism (49) and serotonergic system (50).

In conclusion, our study shows that the *PTPN22* rs2476601 and *CTLA4* rs3087243 autoimmunity risk variants are more frequent in patients with ITO ME/CFS. This finding provides further evidence that there is a genetic predisposition for ME/CFS. The associations with *PTPN22* and *CTLA4* SNPs point to a role of autoreactive T and B cells in the pathomechanism of ME/CFS. In contrast, the lack of association of *CTLA4* and *PTPN22* SNPs and the lower frequencies of *IRF5* and *TNF* risk variants in ME/CFS patients without ITO suggest that the pathomechanism is distinct. These associations need to be confirmed in other ME/CFS patient cohorts. Our findings prompt us to intensify research into autoimmune mechanisms and perform clinical studies with drugs targeting autoreactive B cells.

## Data Availability Statement

The SNP data has been deposited to ClinVar - SCV001167677 and SCV001167678. Other raw data supporting the conclusions of this article will be made available by the authors, without undue reservation, to any qualified researcher.

## Ethics Statement

The studies involving human participants were reviewed and approved by Ethics Committee of Charité-Universitätsmedizin Berlin EA4-090-10. The patients/participants provided their written informed consent to participate in this study.

## Author Contributions

SS and SCB performed the research. SL, JH, FS, AS, and SB participated in data analysis. CS and PG contributed patient material. CS and ML designed the research project. SS and CS wrote the paper. All authors provided final approval of the version to be published.

### Conflict of Interest

AS is employed by the company Labor Berlin—Charité Vivantes GmbH, and ML is employed by Carl-Thiem-Klinikum Cottbus GmbH. The remaining authors declare that the research was conducted in the absence of any commercial or financial relationships that could be constructed as a potential conflict of interest.

## Supplementary Material

Supplementary Table 1 Genotype Counts.xlx.

Supplementary Table 2 Soluble immune markers and lymphocytes for ME/CFS patients with or without ITO.

Supplementary Table 3 Soluble immune markers and lymphocytes for ME/CFS according to non-risk and risk genotype for *PTPN22* rs2476601.

Supplementary Table 4 Soluble immune markers and lymphocytes for ME/CFS according to non-risk and risk alleles for *CTLA4* rs3087243.
