# Supplementary material for: Autoimmunity-Related Risk Variants in PTPN22 and CTLA4 Are Associated With ME/CFS With Infectious Onset
Source: Front Immunol. 2020 Apr 9;11:578. doi: 10.3389/fimmu.2020.00578 (PMC7161310; doi:10.3389/fimmu.2020.00578)
Supplement: Supplementary Table 2 — Soluble immune markers and lymphocytes for ME/CFS patients with or without ITO. [file Data_Sheet_1.docx]

**Supplementary Table 2: Soluble immune markers and lymphocytes for ME/CFS patients w/ or w/o ITO.** Median (range) of CrP, T and B cells, soluble IL-2R, TNFα, C3 and C4 complement component [mg/dl] and IgG. Patients were grouped in w/o or w/ ITO. Statistical analysis was performed using Mann-Whitney U test. A p-value ≤ 0.05 was considered as statistically significant.

|  | **w/o ITO** | **w/ ITO** | **p value** |
| --- | --- | --- | --- |
| CrP [mg/dl] | 0.8 (0.3-57.6)  n=69 | 0.5 (0.03-27.4)  n=220 | **0.009** |
| CD4^+^ cells [/nl] | 0.93 (0.36-2.32)  n=68 | 0.85 (0.31-2.58)  n=225 | 0.178 |
| CD8^+^ cells [/nl] | 0.405 (0.13-0.98)  n=68 | 0.42 (0.1-1.71)  n=225 | 0.353 |
| CD3^+^ [/nl] | 1.485 (0.63-3.26)  n=68 | 1.4 (0.5-3.57)  n=223 | 0.541 |
| CD19^+^ [/nl] | 0.255 (0.08-0.65)  n=68 | 0.23 (0.05-0.65)  n=224 | 0.054 |
| soluble IL-2R [IU/ml] | 348 (152-991)  n=71 | 329 (111-1187)  n=222 | 0.109 |
| TNFα (by CBA) [pg/ml] | 238 (18-1501)  n=65 | 298 (14-1675)  n=211 | 0.203 |
| TNFα (after 4h LPS) [pg/ml] | 1101 (208-2452)  n=69 | 998 (193-3288)  n=217 | 0.532 |
| C3 [mg/dl] | 1040 (660-1880)  n=65 | 990 (73-1850)  n=212 | 0.082 |
| C4 [mg/dl] | 220 (70-480)  n=65 | 220 (14-450)  n=210 | 0.375 |
| IgG [g/l] | 9.8 (6.43-14.43)  n=71 | 9.94 (1.38-1822)  n=225 | 0.605 |

ITO = infection triggered onset, w/ = with, w/o = without, CrP = C-reactive protein; IL-2R = interleukin 2 receptor, CBA = cytometric bead array, TNFα = tumor necrosis factor α, LPS = lipopolysaccharides, C3 = complement protein 3, C4 = complement protein 4. Bold values indicate statistically significant p-values (p ≤ 0.05).

**Supplementary Table 3: Soluble immune markers and lymphocytes for ME/CFS according to non-risk and risk genotype for *PTPN22* rs2476601.** Median (range) of CrP, T and B cells, soluble IL-2R, TNFα, C3 and C4 complement component [mg/dl] and IgG. Patients were grouped in without (GG) or with (GA/AA) *PTPN22* rs2476601 risk allele. A (risk allele is underlined). Statistical analysis was performed using Mann-Whitney U test. A p-value ≤ 0.05 was considered as statistically significant.

| ***PTPN22* rs2476601 G>A** | **GG** | **GA/AA** | **p value** |
| --- | --- | --- | --- |
| CrP [mg/dl] | 0.6 (0.03-57.6)  n=224 | 0.4 (0.3-27.4)  n=63 | **0.036** |
| CD4^+^ cells [/nl] | 0.89 (0.31-2.58)  n=227 | 0.86 (0.45-1.61)  n=64 | 0.299 |
| CD8^+^ cells [/nl] | 0.4 (0.1-1.71)  n=227 | 0.45 (0.19-0.94)  n=64 | 0.310 |
| CD3^+^ [/nl] | 1.425 (0.5-3.57)  n=226 | 1.41 (0.76-2.43)  n=63 | 0.741 |
| CD19^+^ [/nl] | 0.24 (0.05-0.65)  n=226 | 0.23 (0.09-057)  n=64 | 0.402 |
| soluble IL-2R [IU/ml] | 335.5 (111-991)  n=226 | 323 (145-1187)  n=65 | 0.471 |
| TNFα (by CBA) [pg/ml] | 280 (14-1675)  n=212 | 332 (18-1216)  n=62 | 0.310 |
| TNFα (after 4h LPS) [pg/ml] | 1036 (193-3288)  n=221 | 958 (466-2333)  n=63 | 0.805 |
| C3 [mg/dl] | 1020 (73-1880)  n=214 | 980 (650-1590)  n=61 | 0.159 |
| C4 [mg/dl] | 220 (14-480)  n=213 | 200 (70-440)  n=60 | 0.060 |
| IgG [g/l] | 9.945 (4.21-18.22)  n=228 | 9.755 (1.38-14.61)  n=66 | 0.251 |

ITO = infection triggered onset, w/ = with, w/o = without, CrP = C-reactive protein; IL-2R = interleukin 2 receptor, CBA = cytometric bead array, TNFα = tumor necrosis factor α, LPS = lipopolysaccharides, C3 = complement protein 3, C4 = complement protein 4. Bold values indicate statistically significant p-values (p ≤ 0.05).

**Supplementary Table 4: Soluble immune markers and lymphocytes for ME/CFS according to non-risk and risk alleles for *CTLA4* rs3087243.** Median (range) of CrP, T and B cells, soluble IL-2R, TNFα, C3 and C4 complement component [mg/dl] and IgG. Patients were grouped in without (GG) or with (GA/AA) *CTLA4* rs3087243 risk allele G (risk allele is underlined). Statistical analysis was performed using Mann-Whitney U test. A p-value ≤ 0.05 was considered as statistically significant.

| **CTLA4 rs3087243 G>A** | **GG** | **GA** | **AA** | **p value**  **GG vs. GA** | **p value**  **GG vs. AA** | **p value**  **GA vs. AA** |
| --- | --- | --- | --- | --- | --- | --- |
| CrP [mg/dl] | 0.6 (0.03-8.1)  n=110 | 0.57 (0.3-57.6)  n=146 | 0.7 (0.3-21.7)  n=36 | 0.513 | **0.050** | 0.123 |
| CD4^+^ cells [/nl] | 0.85 (0.31-2.08)  n=112 | 0.89 (0.31-2.58)  n=147 | 0.9 (0.45-2.16)  n=32 | 0.504 | 0.816 | 0.922 |
| CD8^+^ cells [/nl] | 0.425 (0.17-1.71)  n=112 | 0.41 (0.1-1.11)  n=147 | 0.385 (0.13-0.91)  n=32 | 0.240 | *0.073* | 0.238 |
| CD3^+^ [/nl] | 1.37 (0.5-2.89)  n=111 | 1.435 (0.63-3.57)  n=146 | 1.415 (0.72-3.18)  n=32 | 0.847 | 0.413 | 0.433 |
| CD19^+^ [/nl] | 0.24 (0.05-0.57)  n=112 | 0.23 (0.08-0.65)  n=146 | 0.245 (0.08-0.65)  n=32 | 0.921 | 0.637 | 0.623 |
| soluble IL-2R [IU/ml] | 328 (111-624)  n=112 | 339.5 (152-1187)  n=148 | 331 (173-804)  n=31 | 0.325 | 0.824 | 0.685 |
| TNFα (by CBA) [pg/ml] | 288 (32-1061)  n=107 | 290.5 (15-1675)  n=136 | 266 (14-705)  n=31 | 0.503 | 0.348 | 0.225 |
| TNFα (after 4h LPS) [pg/ml] | 1090 (208-3009)  n=108 | 999.5 (193-2507) n=144 | 923 (507-3288)  n=32 | 0.152 | 0.309 | 0.898 |
| C3 [mg/dl] | 980 (73-1580)  n=105 | 1025 (123-1850)  n=140 | 1035 (740-1880)  n=30 | 0.841 | 0.109 | 0.182 |
| C4 [mg/dl] | 200 (14-450)  n=105 | 220 (35-440)  n=139 | 250 (150-480)  n=29 | 0.138 | **0.004** | **0.035** |
| IgG [g/l] | 9.805 (5.62-16.79)  n=112 | 10 (1.38-15.7)  n=149 | 9.8 (6.35-18.22)  n=33 | 0.946 | 0.878 | 0.778 |

ITO = infection triggered onset, w/ = with, w/o = without, CrP = C-reactive protein; IL-2R = interleukin 2 receptor, CBA = cytometric bead array, TNFα = tumor necrosis factor α, LPS = lipopolysaccharides, C3 = complement protein 3, C4 = complement protein 4. Bold values indicate statistically significant p-values (p ≤ 0.05).
